# Search of New Tools for Weed Control Using *Piptocarpha rotundifolia*, a Dominant Species in the Cerrado

**DOI:** 10.1021/acs.jafc.1c01880

**Published:** 2021-07-30

**Authors:** Simoni Anese, Carlos Rial, Rosa M. Varela, Ascensión Torres, José M. G. Molinillo, Francisco A. Macías

**Affiliations:** † Federal Institute of Education, Science and Technology of Mato Grosso, Campus Campo Novo do Parecis, MT 235 Km 12, Campo Novo do Parecis, MT 78360-000, Brazil; ‡ Allelopathy Group, Department of Organic Chemistry, Institute of Biomolecules (INBIO), Campus de Excelencia Internacional (ceiA3), School of Science, University of Cadiz, C/ República Saharaui no 7, Puerto Real, Cadiz 11510, Spain

**Keywords:** allelochemicals, piptocarphin A, isolation, phytotoxicity, weed control

## Abstract

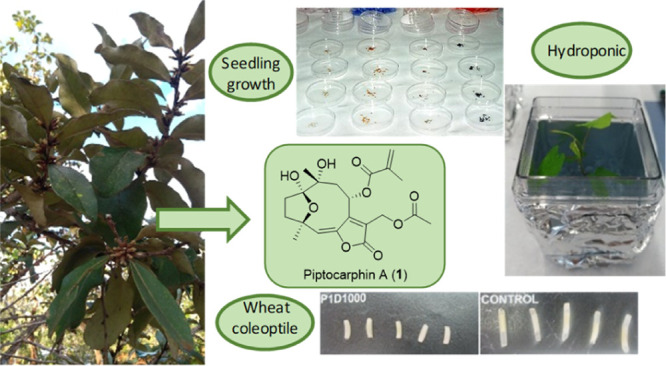

*Piptocarpha rotundifolia* (Less.) Baker stands out as one of the species with the highest frequency, density, and relative dominance in the Cerrado formations. However, no phytochemical studies have been carried out with this species to date. The aim of this study was to evaluate the phytotoxic activity of *P. rotundifolia* leaves in the search of new environmentally friendly tools for weed control. Thus, a wheat coleoptile and phytotoxic bioassay, using relevant agricultural weeds, was used to identify the most active extracts and fractions. The subsequent purification process allowed the isolation of 11 compounds, the phytotoxicity of which was evaluated in terms of wheat coleoptile elongation and with the most sensitive weeds. Piptocarphin A was found to be the major compound and the most active. To confirm its phytotoxic potential, the effect on *Ipomea grandifolia* grown in a hydroponic culture and on metaxylem cells was studied. The results obtained in this study demonstrate that the inhibitory activity displayed by *P. rotundifolia* leaf extract is mainly due to the presence of piptocarphin A. The phytotoxicity shown by *P. rotundifolia* leaf extract, and the isolated compounds, on weeds could provide new tools for weed control in agricultural fields.

## Introduction

Among the world’s important biomes, the Cerrado is considered to be one of the most diverse Savanna on the planet, with more than 12,000 plant species.^1^ Moreover, it has a high level of endemism, with about 44% of the Cerrado’s plant species being endemic.^2,3^ As such, more studies are needed to identify useful Cerrado plants. The lack of knowledge about its biodiversity and possibilities is intensified by being under increasing pressure due to the expansion of agricultural frontiers in the Cerrado regions.^4^ Indeed, a total of 480 endemic species are expected to disappear if vegetation continues to change as a result of agricultural expansion,^5^ thus meaning that, once extinct, the natural resources currently present will be unavailable for subsequent exploitation. Among these, it is important to consider the discovery of natural compounds with chemical structures useful for the synthesis, for example, of natural pesticides.

Asteraceae Brecht. and J. Presl (Compositae), which is the largest family among the Angiosperms, includes 1600–1700 genera and 24,000–30,000 species, thus representing about 10% of all the world’s flora.^6^ In Brazil, the family is represented by 290 genera and 2064 species, which are found in all the biomes, although with greater richness in the Cerrado domain, where a large number of species present high endemism.^7^ Asteraceae are chemically characterized by their marked ability to biosynthesize secondary metabolites with great structural diversity and a wide variety of biological activities. Among these secondary metabolites, terpenoids (mainly sesqui, di, and triterpenes and sesquiterpene lactones) and phenolic compounds (flavonoids and *trans*-cinnamic acid derivatives) are the most representative classes of compounds.^8−10^ Sesquiterpene lactones, which are the most studied group, are almost exclusive to this family and are used as chemotaxonomic markers.^11^ They have been identified in several species of this family such as *Cynara cardunculus*(12,13) or sunflower.^14^ In addition, they exhibit a broad spectrum of biological activity, including allelopathy.^15,16^

*Piptocarpha* R. Br. (Asteraceae) is a neotropical genus that extends from southern Brazil and northern Argentina to Central America and includes approximately 50 species of shrubs and trees.^17^ The tree species are widely distributed throughout the southern Brazilian plateaus. One exception is *Piptocarpha rotundifolia* (Less.) Baker, a tree that is exclusive to the Cerrado domain of central Brazil.^18^ In phytosociological studies, *P. rotundifolia* stands out as one of the species with the highest frequency, density, and relative dominance in the Cerrado formations.^19^

Only few phytochemical studies have been carried out with plants from the genus *Piptocarpha* and none with *P. rotundifolia*. Previous studies with this genus tended to report the isolation of triterpenes, flavonoids, steroids,^20,21^ and sesquiterpene lactones.^22^ The latter, referred to as piptocarphins A-F, demonstrated cytotoxic activity in cancer cells.^22^ For *P. rotundifolia*, there are reports that the leaf extract presented molluscicidal activity,^23^ efficacy against *Aedes aegypti* larvae,^24^ and phytotoxic activity.^25^ The wide distribution and abundance of *P. rotundifolia*, and the lack of studies from a phytochemical and biological point of view, justify the importance of studies that may provide some understanding of the potential use of this species for weed control.

Allelopathy is the science that deals with the study of positive and negative interactions among organisms that are caused by the action of chemical compounds referred to as allelochemicals.^26^ The study of ecological interactions between plants, such as allelopathy, may represent a useful perspective for the development of new classes of environmentally sustainable herbicides.^27^ Indeed, allelochemicals such as sesquiterpene lactones have been used as models for the development of natural herbicides. Natural products are an important alternative due to their great diversity of chemical structures and possibilities of biological properties.^16^

The aim of this study was to evaluate the phytotoxic activity of *P. rotundifolia* leaves in the search of new environmentally friendly tools for weed control that could be used as natural herbicide models in the future. The presence of phytotoxic compounds could also explain the dominance of this species in the Cerrado biome.

## Material and Methods

### General Experimental Procedures

400, 500, and 600 MHz spectrometers (Agilent, Palo Alto, CA, USA) were used to acquire nuclear magnetic resonance (NMR) spectra. The chemical shift relative to the residual 1H signal is given in ppm of CDCl_3_ (δ 7.25) and MeOD (δ 3.33); ^13^C signals are referenced to the solvent signals at δ 77.00 and 49.0 ppm. Ultrasonic extraction was performed using an ultrasonic bath (360 W, JP Selecta, Barcelona, Spain). Silica gel 0.060–0.200, 60A from Acros Organics (Geel, Belgium) or LiChroprep RP 18 (40–63 μm) from Merck (Darmstadt, Germany) was used for column chromatography. High-performance liquid chromatography (HPLC; Merck-Hitachi, Tokyo, Japan) with a refractive index detector was used (Elite LaChrom RI L-2490). A semipreparative column (250 mm × 10 mm i.d., 10 μm LiChrospher 100 RP-18; Merck, Darmstadt, Germany) with a guard column (LiChrospher RP-18; Merck, Darmstadt, Germany) and a LiChrospher Si60 column (250 mm × 10 mm i.d., 10 μm; Merck) with a LiChrospher Si60 guard column (Merck) were used for HPLC.

Chloroform, *n*-hexane, methanol, dichloromethane, ethyl acetate, acetonitrile, and acetone for HPLC were purchased from VWR International (Radnor, PA, USA). MagniSolv chloroform-D1 and CD_3_OD (deuteration degree min. 99.8%) for NMR spectroscopy were purchased from Merck. Water was type I and obtained from an Ultramatic system from Wasserlab (Barbatain, Spain).

### Plant Material

Visibly disease-free *P. rotundifolia* leaves were collected in July 2017 from the Cerrado reserve area in the Federal Institute of Education, Science and Technology of Mato Grosso (IFMT), in the city of Campo Novo do Parecis, Mato Grosso state (Brazil, 13° 40′ S, 57° 53′ W, 572 m a.s.l.). The region is characterized by an Aw climate,^28^ with dry winters (April to September) and wet summers (October to March). One example of the species was sent to the Biology Institute at the Federal University of Uberlandia (UFU) to confirm identification, which was performed by the taxonomist Jimi Naoki Nakajima. A voucher specimen has been deposited in the herbarium at the same institution with the registration code HUFU71960. After collection, the leaves were dried at 40 °C for 72 h and ground into a powder with an industrial mill. This material was used in the study.

*Amaranthus viridis* L. and *Urochloa decumbens* (Stapf) R.D. Webster were purchased from Agro Cosmos (Engenheiro Coelho, Brazil). *Echinochloa crus-galli* (L.) Beauv., *Ipomea grandifolia* (Dammer) O'Donell, *Eragrostis plana* Nees, *Panicum maximum* Jacq., *Lolium perenne* L., and *Lolium rigidum* Gaudin seeds were purchased from Herbiseed (Reading, U,K).

### Phytochemical Study

#### Initial Extraction

**Figure 1:**
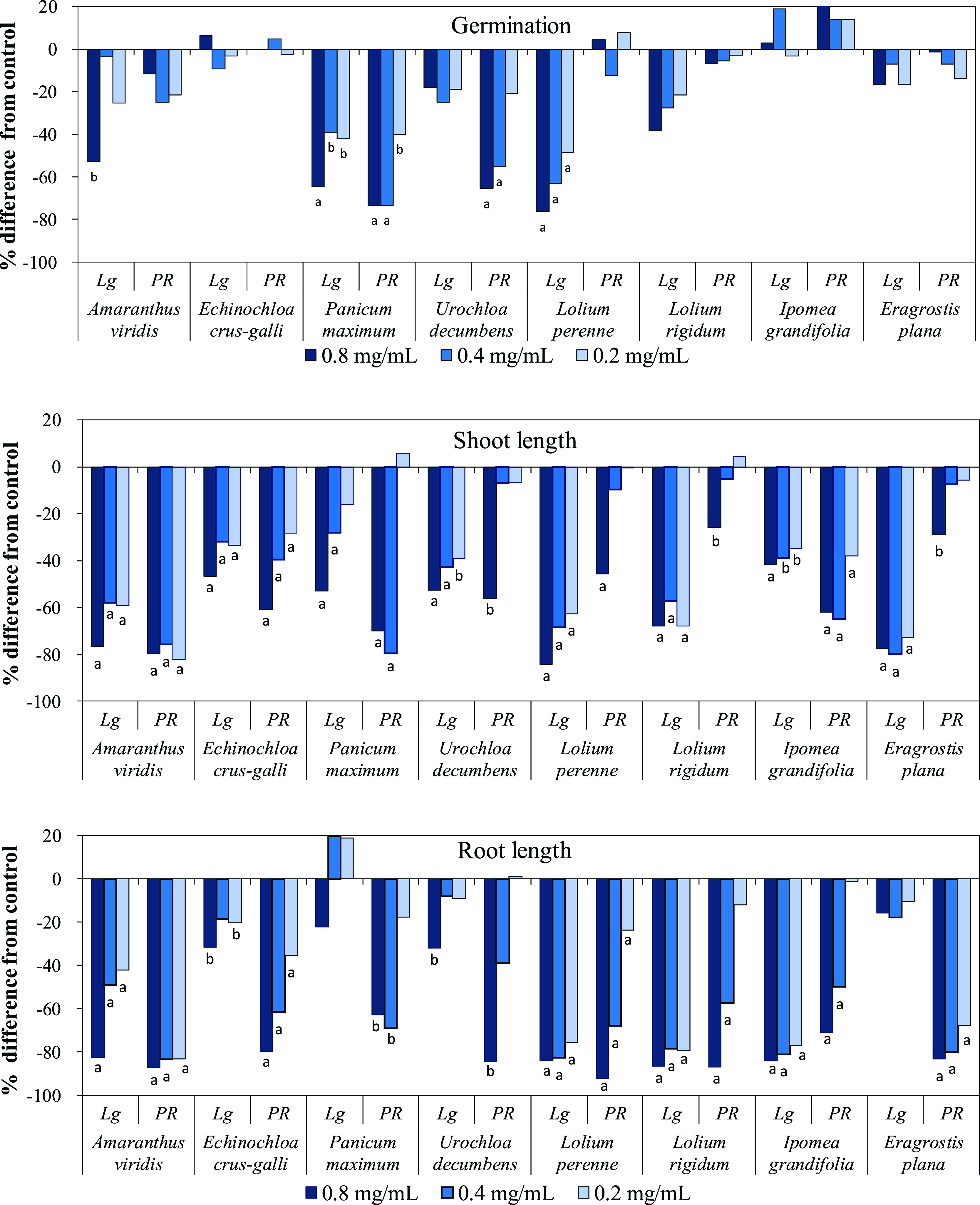
Effects of the herbicide Logran (Lg) and the EtOAc fraction from *Piptocarpha rotundifolia* (PR) leaves on growth and germination of weed species. Values are expressed as percentage difference with respect to the control. Significance levels *p* < 0.01 (a) or 0.01 < *p* < 0.05 (b).

An amount of 1.6 kg of leaf powder was subjected to aqueous extraction, in portions of 100 g of plant powder in 500 mL of distilled water, in an ultrasonic bath for 10 min (procedure repeated twice). The resulting aqueous extract was transferred to a separating funnel, in 1000 mL portions, and partitioned with ethyl acetate (EtOAc) (3 × 1000 mL) to give aqueous and EtOAc fractions. The EtOAc fraction was concentrated in a rotary evaporator, at low pressure and 37 °C, and an aliquot of the initial aqueous extract (reserved previously) and the aqueous fraction were evaporated in a Savant model Speedvac SC210 vacuum centrifuge system to dryness. Finally, the activity of the initial aqueous extract and the EtOAc and aqueous fractions was evaluated in wheat coleoptile bioassays (S3). As the EtOAc fraction was found to be the most active, its activity was assayed for phytotoxicity on weeds (Figure 1).

#### Purification of the EtOAc Fraction

Based on the initial bioassay results, the EtOAc fraction was selected to continue the phytochemical study. For this purpose, 15 g of the fraction was subjected to reverse-phase column chromatography (filled with RP-18 silica), eluting with a H_2_O/methanol (MeOH) mixture (from 100:0 to 0:100 v/v, with a 20% increase; 750 mL of each polarity) and, finally, with dichloromethane to remove chlorophylls. After thin-layer chromatography analysis, four fractions of interest were obtained: A (100% H_2_O + 20% MeOH, 1.39 g), B (40% H_2_O + 60% MeOH, 7.1 g), C (80% MeOH, 2.8 g), and D (100% MeOH, 1.27 g). Evaluation using wheat coleoptiles bioassay showed that fractions B, C, and D exhibited high inhibitory activity (S4) and, as such, these fractions were separated using chromatographic techniques for the isolation of active compounds.

Fraction B (7.1 g) was chromatographed on a silica gel column, eluting with a chloroform (CHCl_3_)/MeOH gradient from 100:0 to 0:100 v/v, with a 10% increase, 500 mL of each polarity, to yield 14 sub-fractions (B1–B14). Sub-fraction B2 (1.4 g) was identified as compound **1**, which was isolated pure and considered to be the major compound in the plant. Sub-fraction B4 (1.3 g) was purified by HPLC (semipreparative column), using the solvents hexane/EtOAc (isocratic method 35:65 v/v, flow 3 mL min^–1^), to give compound **2** (556 mg). Sub-fraction B6 (60 mg) was purified by HPLC (RP-18 semipreparative column), eluting with H_2_O/MeOH/acetonitrile (MeCN) (40:30:30 v/v, flow 3 mL min^–1^), to give five sub-fractions (B6.1-B6.5). Sub-fraction B6.2 (69.5 mg) was characterized as compound **2**, and sub-fraction B6.4 (28.3 mg) was purified by HPLC (RP-18 semipreparative column), using the same solvents (50:25:25 v/v, flow 3 mL min^–1^), to yield compound **3** (11.5 mg). Sub-fractions B12 (66 mg) and B14 (434 mg), separately, were purified by HPLC (RP-18 semipreparative column), eluting with H_2_O/MeOH/acetone (63:18:18 v/v, flow 3 mL min^–1^), to give compound **4** (16 mg) and a mixture of compounds **5** and **6** (217 mg), respectively.

Fraction C (2.8 g) was separated on a chromatographic column, eluting with dichloromethane/EtOAc mixtures from 0 to 100% polarity increase in EtOAc (5% increase, 300 mL of each polarity) and ending with 100% MeOH, obtaining 10 sub-fractions (C1–C10). Sub-fraction C3 (103 mg) was purified by HPLC (semipreparative column), using hexane/EtOAc as solvent (isocratic method 70:30 v/v, flow 3 mL min^–1^), to give compound **7** (44.1 mg). Sub-fraction C6 (40 mg) was purified by HPLC (RP-18 semipreparative column), eluting with H_2_O/MeOH/acetonitrile (MeCN) (40:30:30 v/v, flow 3 mL min^–1^), to give compound **8** (C6.4–8.3 mg). Sub-fraction C7 (60 mg) was purified by HPLC (RP-18 semipreparative column), using the same mixture of solvents and previous proportions, to give compounds **9** (C7.6- 9.4 mg) and **10** (C7.8-8.3 mg). Fraction D (1 g) was chromatographed using hexane/acetone mixtures as the mobile phase, from 0 to 100% increase in acetone (10% increase, 300 mL of each polarity) and ending with 100% MeOH, to yield eight sub-fractions (D1–D8). Subfraction D1 (150 mg) was subjected to further fractionation using a chromatographic column using mixtures of hexane/EtOAc (100:0, 95:5, 90:10, 85:15, and 0:100 v/v; 100 mL of each polarity) as the mobile phase and ending with 100% MeOH, yielding four sub-fractions (D1.1–D1.4). Sub-fraction D1.2 was identified as compound **11** (19.4 mg).

**Figure 2:**
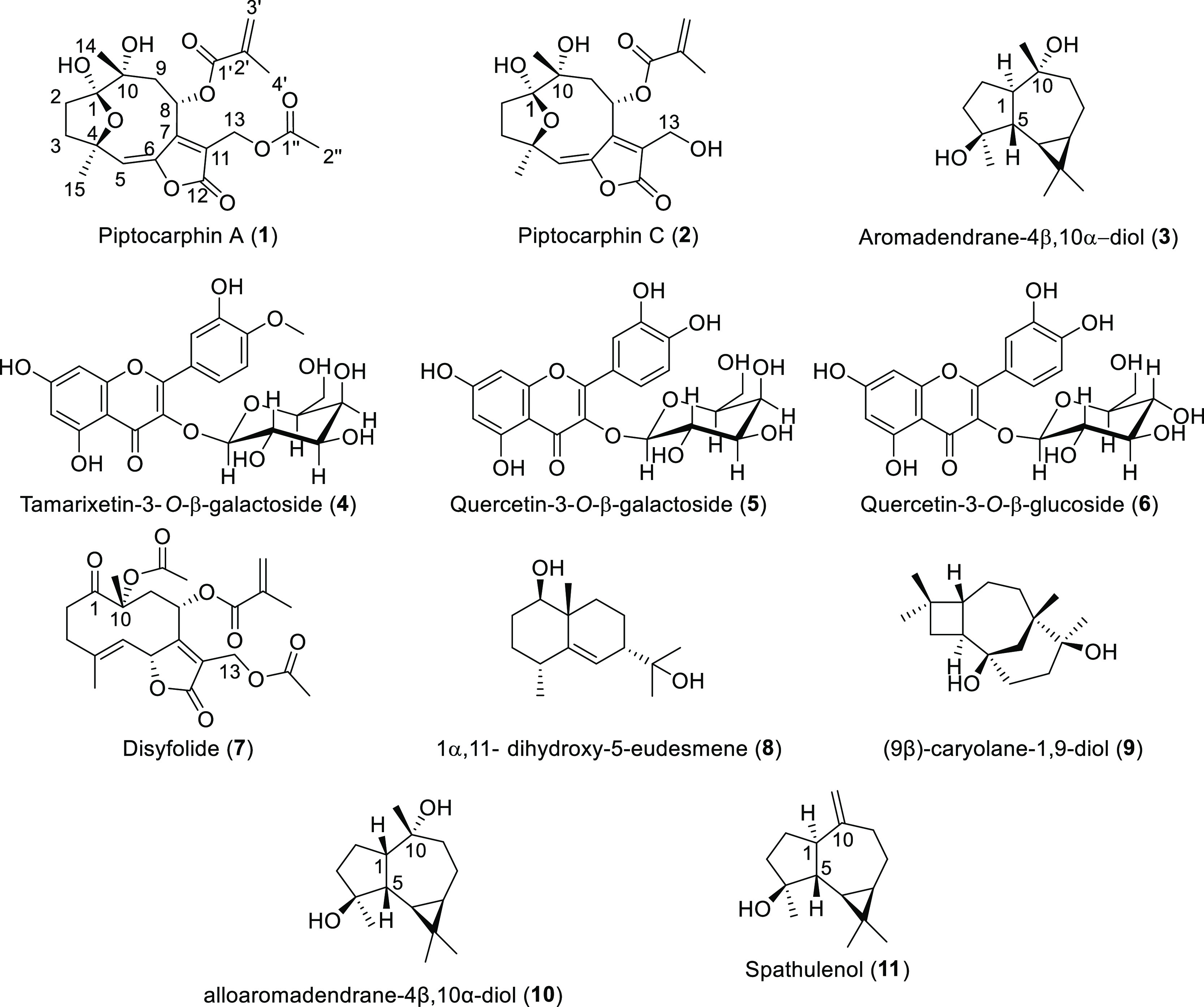
Compounds isolated from *P. rotundifolia* leaves: piptocarphin A (**1**), piptocarphin C (**2**), aromadendrane-4β,10α-diol (**3**), tamarixetin-3-*O*-β-galactoside (**4**), quercetin-3-*O*-β-galactoside (**5**), quercetin-3-*O*-β-glucoside (**6**), disyfolide (**7**), 1α-11-dihydroxy-5-eudesmene (**8**), (9β)-caryolane-1,9-diol (**9**), alloaromadendrane-4β,10α-diol (**10**), and spathulenol (**11**).

The identification of the compounds (Figure 2) was achieved by using the complete physical, spectroscopic, and spectrometric data set obtained. All data correspond with those reported in the literature for the isolated compounds. All the compounds isolated were evaluated in coleoptile bioassays, and the most actives were evaluated in phytotoxicity bioassays.

### Bioassays

#### Wheat Coleoptile Bioassay

The wheat coleoptile bioassay was performed according to the procedure previously described by Rial and co-workers^16^ (S1). Thus, fractions were tested at concentrations of 0.2, 0.4, and 0.8 mg mL^–1^, while isolated compounds were tested at concentrations of 10, 30, 100, and 300 μM and 1 mM. Each evaluated solution maintained a constant dimethyl sulfoxide (DMSO) concentration of 5 μL/mL. Each treatment was tested in triplicate by adding 2 mL of each solution and five fragments of wheat coleoptile to a glass test tube (16 × 100 mm, 10 mL). One control containing the buffer solution with DMSO (5 μL/mL) was included. The commercial herbicide Logran (terbutryn 59.4% and triasulfuron 0.6%) was used as an internal reference, and its concentration and conditions were the same as previously reported. Welch’s test was performed for statistical analysis of the data and to express it as a percentage difference relative to the control. Positive values represent stimulation, and negative values represent inhibition.

#### Phytotoxicity Bioassay

The ethyl acetate fraction and the most active compounds were assessed for phytotoxic activity following the procedure previously described by Rial and co-workers^16^ (S2) on eight agricultural weeds, which were selected because they belong to and represent important families in agricultural weeds: morning glory (*I. grandifolia*, Convolvulaceae), barnyard grass (*E. crus-galli*, Poaceae), slender amaranth (*A. viridis*, Amaranthaceae), guinea grass (*P. maximum*, Poaceae), brachiaria (*U. decumbens*, Poaceae), perennial ryegrass (*L. perenne*, Poaceae), annual ryegrass (*L. rigidum*, Poaceae), and capim-annoni (*E. plana*, Poaceae). In addition, the invasive grasses *P. maximum*, *E. crus-galli,* and *E. plana*, evaluated in the present study, are considered aggressive invasive species in Brazil, which, in addition to natural reserves such as Cerrado, harm many agricultural crops.^29,30^ To break the physical dormancy, *I. grandifolia* seeds were immersed in a concentrated sulfuric acid solution for 5 min. After that, they were washed in water for use in the tests^31^

Solutions containing the fractions and the isolated compounds were tested at the same concentrations and conditions reported above. At all the concentrations tested and in the control, a constant concentration of DMSO (5 μL/mL) was ensured.

The experimental design was completely randomized and contained four replicates of 20 seeds for each concentration. The experiment was conducted in a germination chamber at 25 °C with a 12 h photoperiod for 7 days for *I. grandofolia*(31) and at 25 °C in the dark for the other species. Bioassays took 6 days for *E. plana*, *A. viridis*, *E. crus-galli*, *L. perenne*, and *L. rigidum* and 8 days for *P. maximum* and *U. decumbens*. After growth, plants were frozen at −10 °C for 24 h. The germination ratio, root length, and shoot length were recorded using a Fitomed system.^32^ The data were analyzed statistically using Welch’s test, with significance fixed at 0.01 and 0.05. The germination ratio, root length, and shoot length are presented as percentage differences with respect to the control. Zero represents control, positive values represent stimulation, and negative values represent inhibition.

#### Hydroponic Bioassay

For the hydroponic bioassay with the most active compound, *Ipomea gradifolia* seedlings were selected and grown previously in pots with a volume of 250 mL, filled with 200 g of washed river sand, vermiculite, and clay in a ratio of 1:1:1. Four seeds were sown in each pot, with 4 replicates per treatment. Ten days after emergence of the seedlings, water was replaced with nutrient solution as described by Hoagland and Arnon^33^ at 1/3 ionic strength for acclimation of the seedlings, which were then transferred, 20 days post-emergence, to glass containers with a diameter of 6 cm and height of 15 cm containing solutions of the evaluated compound at different concentrations and wrapped externally with aluminum foil. As a substrate, 200 g of 3 mm diameter glass beads was used and 40 mL of each solution was added per container. This process ensures the germination of the seeds and allows us to select homogeneous-growth seeds and also allows us to control the concentration of the tested compound.

The pure compound was pre-dissolved in DMSO (5 μL/mL solution) and diluted in nutrient solution as described by Hoagland and Arnon^33^ at 1/3 ionic strength and subsequently dissolved at concentrations of 1000, 300, 100, 30, and 10 μM. As a control, Hoagland and Arnon^33^ solution at 1/3 ionic strength plus DMSO was used. Four replicates were performed for each concentration. The ends of the containers were capped with Parafilm in order to maintain moisture. The experiment was conducted for 20 days in a climatic chamber regulated at 25 °C with a photoperiod of 12 h. After that, the dry biomass of roots and shoots was obtained by collecting the material and packing it in paper bags followed by drying in an air circulation oven at 65–70 °C to constant mass. The results are shown as percentage differences compared to the control, as described in phytotoxic bioassay. The data were analyzed statistically using Welch’s test, with significance fixed at 0.01 and 0.05.

#### Analysis of Metaxylem Elements

*I. grandifolia* seedlings grew at different concentrations of the most active compound isolated from *P. rotundifolia* and the control for four days, under the same conditions tested in the phytotoxic study. After that, the primary root segments of the seedlings were removed and subjected to a modified Fuchs staining method.^34^ Briefly, the roots were immersed in 70% alcohol for five days and placed in a solution of 10% NaOH at 60 °C for 48 h to complete clarification. The root segments were subsequently immersed in the safranin reagent (C_20_H_19_N_4_Cl) and 10% NaOH, for 24 h at 60 °C, as reported by Anese et al.^31^ After staining, the roots were mounted on glass slides with Apathy’s syrup^31,35^ for observation under an Axio optical microscope coupled to a camera (ZEISS Axiocam ERs 5s Ver 5.0). Four primary roots of *I. grandifolia* seedlings grown at different concentrations of the compound and control were assessed. Half the length of each root from the mature region (central) toward the stem separation was photographed. From each image, the size of 15 metaxylem vessel elements was evaluated, at 20× magnification,^31^ with the aid of ZEN 2 (blue edition) software. Analysis of variance (ANOVA) followed by Scott–Knott’s test at a significance level of 0.05 was performed for the data obtained.

### Statistical Analysis

IC_50_ values for the activity inhibition data were determined by performing a non-linear regression with the GraphPad Prism 5 package (San Diego, CA, USA). Cluster analyses were performed using Statistica v.7.0 software (Tulsa, OK, USA). Squared euclidian distances and complete linkage were used for the analysis.

## Results and Discussion

A bioguided isolation was carried out to study the phytotoxic activity of *P. rotundifolia*. To that end, dry and crushed *P. rotundifolia* leaves were submitted for aqueous extraction and an aliquot of the resulting extract was partitioned by liquid–liquid extraction with EtOAc, resulting in the aqueous and EtOAc fractions. Aliquots of the crude aqueous extract and each fraction were evaporated to dryness and used in wheat coleoptile bioassays. The EtOAc fraction caused 100% inhibition in the elongation of wheat coleoptiles at concentrations of 0.8 and 0.4 mg mL^–1^. No inhibitory activity was recorded for the aqueous extract or the aqueous fraction (S3). Similar results have been observed in previous studies, which show the ethyl acetate fraction being much more active than the original aqueous extract and the aqueous fraction.^36^ The relevant inhibitory effect on wheat coleoptiles caused by the EtOAc fraction indicates that it has bioactive compounds with a phytotoxic effect. Subsequently, the remaining crude aqueous extract was partitioned with EtOAc to provide a larger EtOAc fraction, yielding 15 g (0.9%). The phytotoxicity levels of this fraction were evaluated using weed seeds (*I. grandifolia*, *E. crus-galli*, *A. viridis*, *P. maximum*, *U. decumbens*, *L. perenne*, *L. rigidum,* and *E. plana*) at the same concentrations as for the coleoptile wheat bioassay (Figure 1). All of these weeds are problematic in agricultural crops.^37^ In addition, the invasive grasses *P. maximum*, *E. crus-galli,* and *E. plana* are considered aggressive invasive species in Brazil and identified as barriers to natural regeneration and establishment of native species in the Cerrado biome,^29,30^ where the species *P. rotundifolia* is widely recorded.

The EtOAc fraction significantly inhibited seedling growth. Indeed, it was observed that this fraction was active in the inhibition of root and shoot growth of all species, with more pronounced effects at the highest concentration evaluated and an evident decrease in activity levels with dilution (Figure 1). In general, the inhibitory effect of the EtOAc fraction was more pronounced for primary root growth than for shoot growth of the target weed seedlings, thus corroborating other studies that evaluated the effect of extracts and fractions from allelopathic plants on weeds.^35,36^ Root growth is characterized by high metabolic rates, and for this reason, roots are highly susceptible to environmental stresses, such as allelochemicals in the substrate.^38^ Based on the IC_50_ values (Table 1), *A. viridis* was the most affected weed in terms of both root and shoot growth (0.042 and 0.045 mg mL^–1^, respectively). Regarding germination, the extract showed inhibitory activity on *P. maximun* and *U. Decumbens*, with activities higher than those of Logran.

**Table 1:** IC_50_ Values for the EtOAc Fraction from *Piptocarpha rotundifolia* (PR) Leaves and the Herbicide Logran on Root and Shoot Growth of Weed Species

	root				shoot			
	PR		Logran		PR		Logran	
	IC_50_ (mg mL^–1^)	*R*^2^	IC_50_ (mg mL^–1^)	*R*^2^	IC_50_ (mg mL^–1^)	*R*^2^	IC_50_ (mg mL^–1^)	*R*^2^
*Amaranthus viridis*	0.042	0.99	0.308	0.97	0.045	0.97	0.189	0.97
*Echinochloa crus-galli*	0.286	0.99	a		0.562	0.99	0.843	0.97
*Panicum maximum*	0.376	0.91	a		0.390	0.75	0.839	0.99
*Uruchloa decumbens*	0.514	0.86	a		a		0.557	0.96
*Lolium perenne*	0.284	0.92	0.061	0.99	a		0.135	0.99
*Lolium rigidum*	0.376	0.90	0.056	0.99	a		0.143	0.91
*Ipomea grandifolia*	0.515	0.90	0.060	0.99	0.301	0.97	0.841	0.95
*Eragrostis plana*	0.093	0.99	a		a		0.072	0.98

a 50% of inhibition was not achieved at the highest concentration.

**Figure 3:**
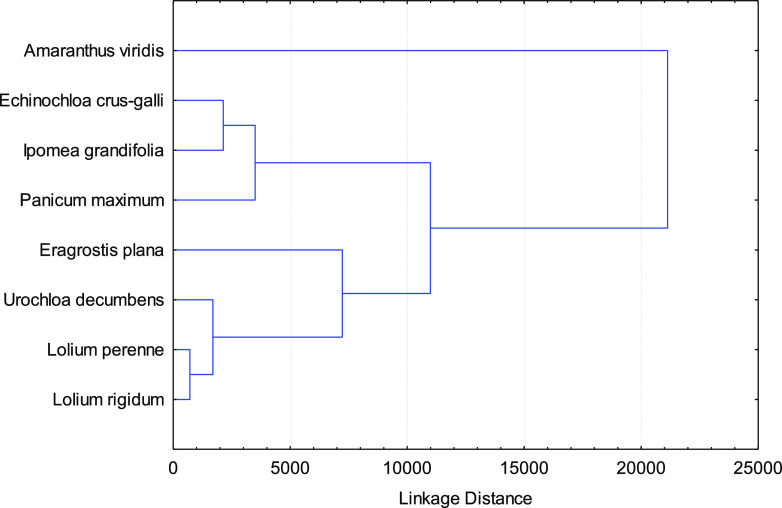
Cluster analysis of the phytotoxic activity of the EtOAc fraction from *Piptocarpha rotundifolia* leaves on shoot and root growth in weed species.

In order to select the most sensitive species for phytotoxic bioassay, a cluster analysis was performed using the phytotoxic bioassay results of the EtOAc fraction from *P. rotundifolia* leaves (Figure 3). This analysis showed that *A. viridis* was the most sensitive species, being grouped independently. In addition, *E. crus-galli*, *I. grandifolia,* and *P. maximum* were selected as the most active at the following level. *E. plana* was also selected because it was grouped independently in the next level.

### Isolation of Phytotoxic Compounds

**Figure 4:**
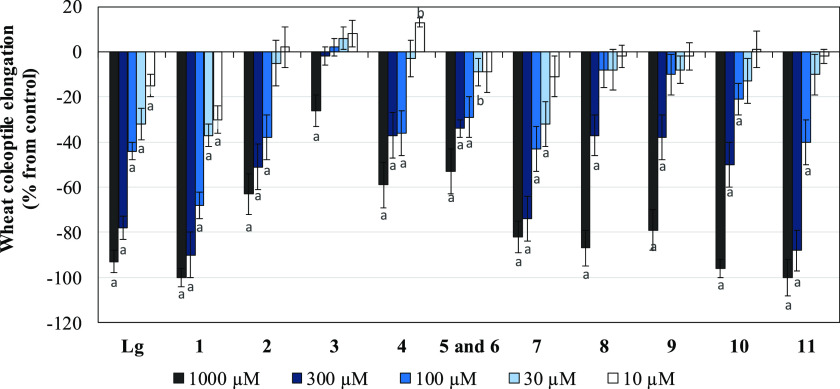
Effect of the herbicide Logran (Lg) and the compounds obtained from *P. rotundifolia* leaves on wheat coleoptile elongation. Values are expressed as percentage difference with respect to the control. Each bar is the mean ± standard deviation. Significance levels *p* < 0.01 (a) or 0.01 < *p* < 0.05 (b).

The initial results showed that *P. rotundifolia* has phytotoxic activity on weed species. Identification of individual components and the evaluation of their bioactivity could facilitate the discovery of new tools for crop protection. Continuing the study, this fraction was subjected to reverse-phase column chromatography (RP-18) to give four fractions of interest, which were evaluated in coleoptile bioassays. Fractions B, C, and D showed strong inhibitory activity, similar to the inhibition caused by the herbicide at the same concentrations (S4). Fractionation of these three fractions led to the isolation of 11 compounds: three sesquiterpene lactones, three flavonol glycosides, and five sesquiterpenoids (Figure 2). The identification of the compounds was achieved by using the complete physical, spectroscopic, and spectrometric data set obtained. All the data correspond with those reported in the literature for piptocarphin A (**1**),^22^ piptocarphin C (**2**),^22^ aromadendrane-4β,10α-diol (**3**),^39^ tamarixetin-3-*O*-β-galactoside (**4**),^40^ quercetin-3-*O*-β-galactoside (**5**),^41^ and quercetin-3-*O*-β-glucoside (**6**),^42^ isolated from fraction B, disyfolide (**7**),^43^ 1α-11-dihidroxy-5-eudesmene (**8**),^44^ (9β)-caryolane-1,9-diol (**9**),^45^ and alloaromadendrane-4β,10α-diol (**10**),^46^ isolated from fraction C, and spathulenol (**11**),^39^ isolated from fraction D. All compounds were isolated and reported for the first time in *P. rotundifolia,* and the phytotoxicity of some of them has been already reported.^47−49^ Their phytotoxic activities were evaluated in coleoptile bioassay (Figure 4).

### Wheat Coleoptile Bioassay with Isolated Compounds

The sesquiterpene lactone piptocarphin A (**1**) caused the highest percentage inhibition in the wheat coleoptile elongation assay, resulting in inhibition of 100 and 90%, respectively, at the concentrations of 1000 and 300 μM (Figure 4). The sesquiterpene spathulenol (**11**) also showed strong inhibition, reaching 98% at the highest concentration evaluated. These values for both compounds were even higher than those achieved using the herbicide Logran. The coleoptile bioassay results allowed us to calculate the IC_50_ value for each product evaluated and to organize the compounds according to their phytotoxicity (Table 2). The activity shown by the compounds correlated with that obtained for fractions in the coleoptile bioassays (S4). Thus, some of the most active compounds, with activity levels similar to, or even higher than, the Logran herbicide were isolated from the most active fractions. For example, piptocarphin A (**1**) and piptocarphin B (**2**) were isolated from fraction B, spathulenol (**11**) from fraction D, and disyfolide (**7**) from fraction C. The sesquiterpenes 1α-11-dihydroxy-5-eudesmene (**8**), (9β)-caryolane-1,9-diol (**9**), and alloaromadendrane-4β,10α-diol (**10**), which showed high inhibition values at 1000 μM, were also isolated from fraction C, although this activity decreased significantly for the other concentrations evaluated (Figure 4). This fast decrease in activity with the dilution has been previously reported for other sesquiterpenes.^16,50^

**Table 2:** IC_50_ Values for the Herbicide Logran and the Compounds Obtained from *P. rotundifolia* Leaves on Wheat Coleoptile Elongation

	wheat coleoptile	
	IC_50_ (μM)	*R*^2^
**1**	48.4	0.99
**L**ogran	105.0	0.99
**7**	105.9	0.98
**11**	116.9	0.97
**2**	239.6	0.96
**10**	267.7	0.97
**4**	380.6	0.93
**8**	414.4	0.97
**9**	439.3	0.98
**5** and **6**	784.3	0.97
**3**	a	

a 50% inhibition was not achieved at the highest concentration.

In view of the results, a SAR study could be carried out. First, sesquiterpenes have shown higher activity than flavones. In addition, sesquiterpene lactones seem to be a bit more active. In the case of piptocarphins and disyfolide, acetylation of the hydroxyl group at C13 increases substantially their phytotoxicity. However, hydroxyl groups at positions C1 and C10 increase the activity. Regarding compounds 3, 10, and 11, a SAR study is ambiguous. On one hand, comparing 3 and 11, the presence of the double bond at C10 instead of a hydroxyl group seems crucial for the bioactivity. However, on the other hand, comparing 3 and 10, we can conclude that the configuration of C1 and C5 is also involved in their phytotoxicity.

### Phytotoxic Bioassay of the Most Active Compounds

The compounds selected were **1**, **2**, **7**, **10,** and **11** for their higher activity in wheat coleoptile bioassays and according to the IC50 values (Table 2). However, **10** could not be evaluated due to the low quantity isolated. They were evaluated in phytotoxicity bioassays of the most sensitive weed seeds selected using the cluster analysis in Figure 3, namely, *A. viridis*, *E. crus-galli*, *I. grandifolia*, *P. maximum,* and *E. plana*.

**Figure 5:**
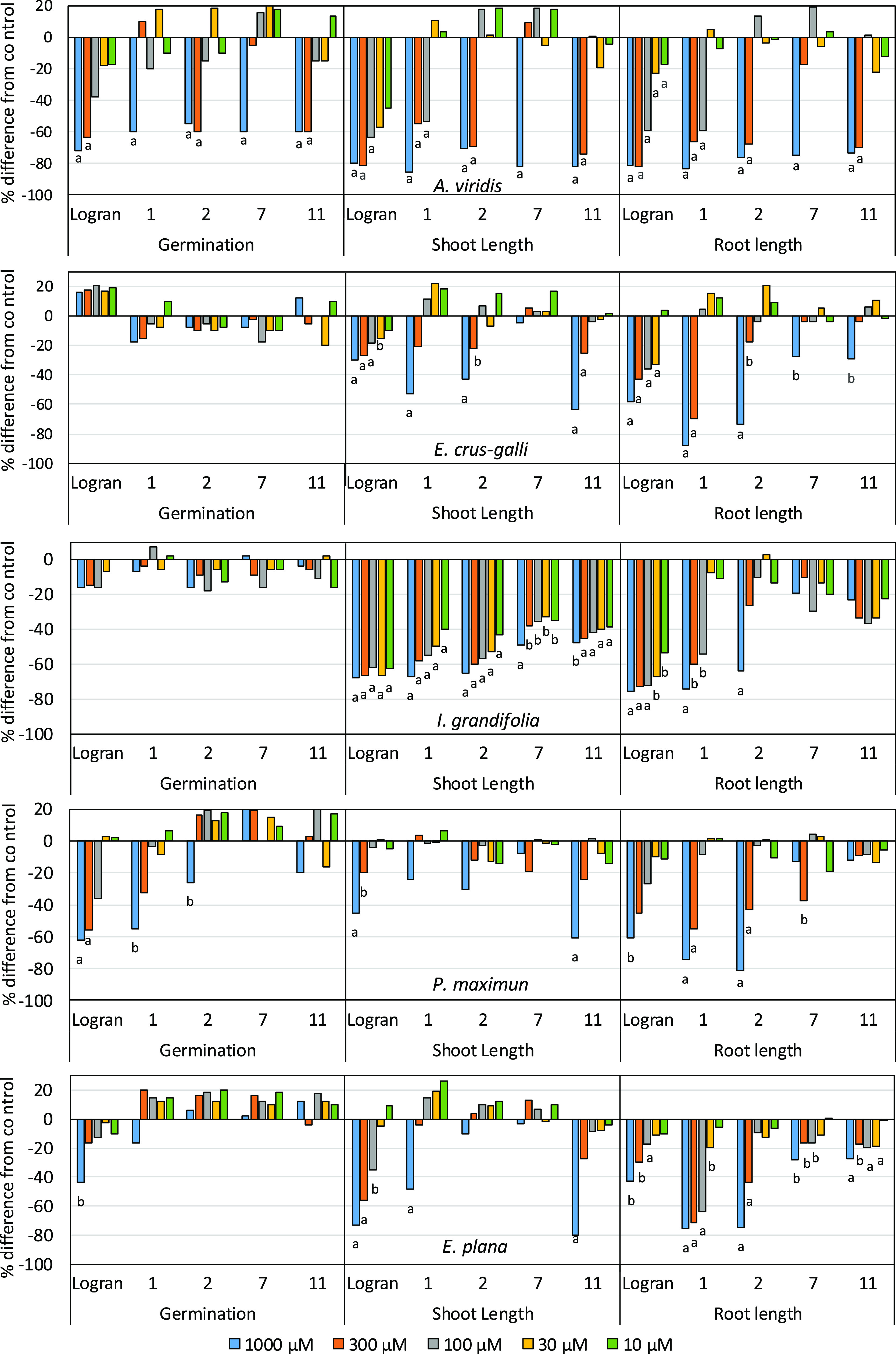
Effect of the herbicide Logran (Lg) and the compounds obtained from *P. rotundifolia* leaves on the germination and growth of weeds. Values are expressed as percentage difference with respect to the control. Significance levels *p* < 0.01 (a) or 0.01 < *p* < 0.05 (b).

Seed germination was the parameter least affected by the compounds studied. Piptocarphin A (**1**) was active on *P. maximum* germination, with an inhibition similar to that for the herbicide (60% inhibition) at a concentration of 1000 μM. The germination of *A. viridis* was found to be sensitive to the effect of compounds **1**, **2**, **7,** and **11** (Figure 5). For shoot length, *A. viridis* was sensitive to the inhibitory effects of compounds **1** (IC_50_ = 151.5 μM), **2** (IC_50_ = 327.6 μM), and **11** (IC_50_ = 261.0 μM) (Table 3), only at the highest concentrations, and *I. grandifolia* was sensitive to the inhibitory effects of compounds **1** (IC_50_ = 15.0 μM) and **2** (IC_50_ = 10.9 μM). The shoot length of the others weeds was scarcely influenced by these compounds, with inhibition values between 20 and 60% for compounds **1**, **2,** and **11**, depending on the species evaluated (Figure 5).

**Table 3:** IC_50_ Values for the Herbicide Logran and the Compounds Obtained from *P. rotundifolia* Leaves on the Growth of Weeds

	*Eragrostis plana*		*Echinochloa crus-galli*		*Panicum maximum*		*Amaranthus viridis*		*Ipomea grandifolia*	
	shoot IC_50_ μM (*R*^2^)	root IC_50_ μM (*R*^2^)	shoot IC_50_ μM (*R*^2^)	root IC_50_ μM (*R*^2^)	shoot IC_50_ μM (*R*^2^)	root IC_50_ μM (*R*^2^)	shoot IC_50_ μM (*R*^2^)	root IC_50_ μM (*R*^2^)	shoot IC_50_ μM (*R*^2^)	root IC_50_ μM (*R*^2^)
**L**ogran	196.8 (0.98)	a	a	438.2 (0.88)	a	475.0 (0.98)	13.9 (0.93)	71.3 (0.98)	6.1 (0.92)	6.2 (0.93)
**1**	a	69.2 (0.96)	776.0 (0.96)	257.8 (0.91)	a	328.9 (0.97)	151.5 (0.93)	116.0 (0.93)	15.0 (0.81)	129.6 (0.92)
**2**	a	444.4 (0.98)	a	580.5 (0.95)	a	434.0 (0.96)	327.6 (0.87)	330.2 (0.87)	10.9 (0.81)	723.2 (0.97)
**7**	a	a	a	a	a	a	799.2 (0.83)	701.4 (0.90)	a	a
**11**	541.3 (0.97)	a	697.8 (0.99)	a	927.6 (0.96)	a	261.0 (0.85)	318.9 (0.89)	a	a

a 50% inhibition was not achieved at the highest concentration.

Root growth was the most affected parameter in all weeds tested, with the sesquiterpene lactones piptocarphin A (**1**) and piptocarphin C (**2**) showing a stronger inhibitory activity than the other compounds. Piptocarpin A (**1**) was even more active than the herbicide Logran in some weed species (Figure 5 and Table 3).

Fast loss of activity, as previously reported in coleoptile bioassay, was also observed for all parameters and weeds tested with the dilution, except for *I. grandifolia* shoot length, being very active at high concentrations but losing the activity completely at low doses.

To confirm the most active compound, a cluster analysis was carried out using the germination and the root and shoot length of all weeds tested (S5). With regard to the cluster analysis, piptocarphin A (**1**), which was grouped independently, was the most active compound, with piptocarphin C (**2**) and spathulenol (**11**) being grouped together at the second level. Disyfolide (**7**), which was classified far away from piptocarphin A in the cluster, was identified as the least active compound.

To summarize the phytotoxic bioassay, the sesquiterpene lactone piptocarphin A (**1**) was the compound isolated in the largest quantity from *P. rotundifolia* leaves and also the one that contributed most to the phytotoxic activity exhibited by the plant in this study for all parameters evaluated. The second compound isolated in the highest quantities from the plant, piptocarphin C (**2**), also demonstrated good levels of inhibitory effects, particularly on weed root growth. The inhibitory activity of the sesquiterpene spathulenol (**11**) on some species in this study should also be highlighted.

### Hydroponic Bioassay of piptocarphin A (1)

**Figure 6:**
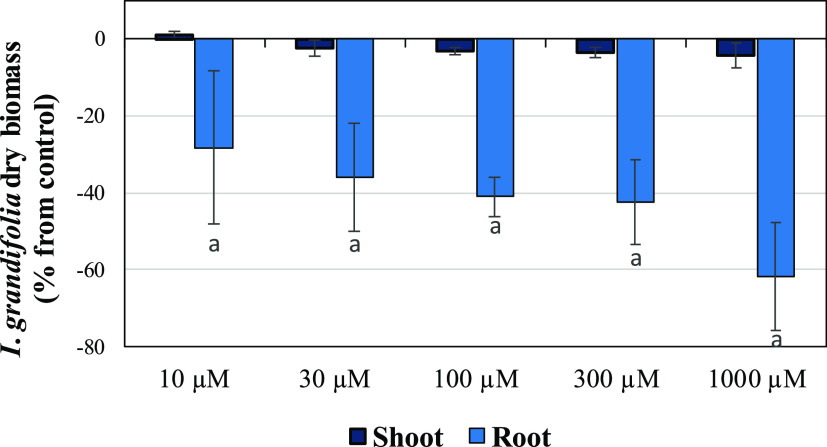
Effect of piptocarphin A obtained from *P. rotundifolia* leaves on the dry shoot and root biomass of *I. grandifolia* grown under hydroponic conditions. Values are expressed as percentage difference with respect to the control. Vertical bars represent standard deviations. Significance levels *p* < 0.01 (a) or 0.01 < *p* < 0.05 (b).

To assess the phytotoxic potential of the most active compounds, piptocarphin A was measured under hydroponic conditions in the next level of bioassays. *I. grandifolia* was chosen for this hydroponic bioassay because compound **1** inhibited its growth at high percentage and low doses, for shoots and roots (Figure 5 and Table 3). Although the biomass accumulation in shoots was not affected by piptocarphin A, the dry weight of *I. grandifolia* roots was significantly reduced, and the dry biomass yield was reduced by 40 and 60% at concentrations of 300 and 1000 μM, respectively (Figure 6). Phytotoxic substances could directly affect the development and accumulation of dry biomass as they interfere with cell division, membrane permeability, and enzyme activity, as reported by da Silva et al.^51^ and Teerarak et al.^52^

### Study of Root Metaxylem Elements

**Figure 7:**
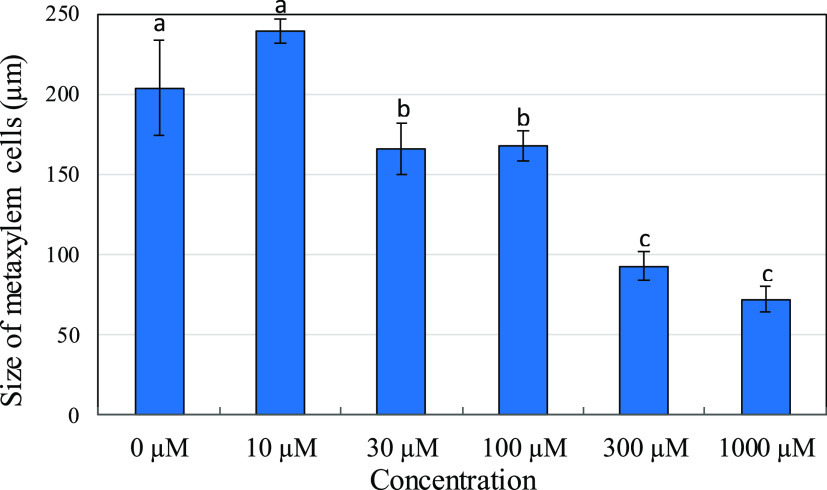
Size of metaxylem cells (μm) in *I. grandifolia* seedlings roots treated with compound **1** (piptocarphin A) at various concentrations. Treatments with different letters differ by Scott–Knott’s test at a probability of 0.05. Vertical bars represent standard deviations.

**Figure 8:**
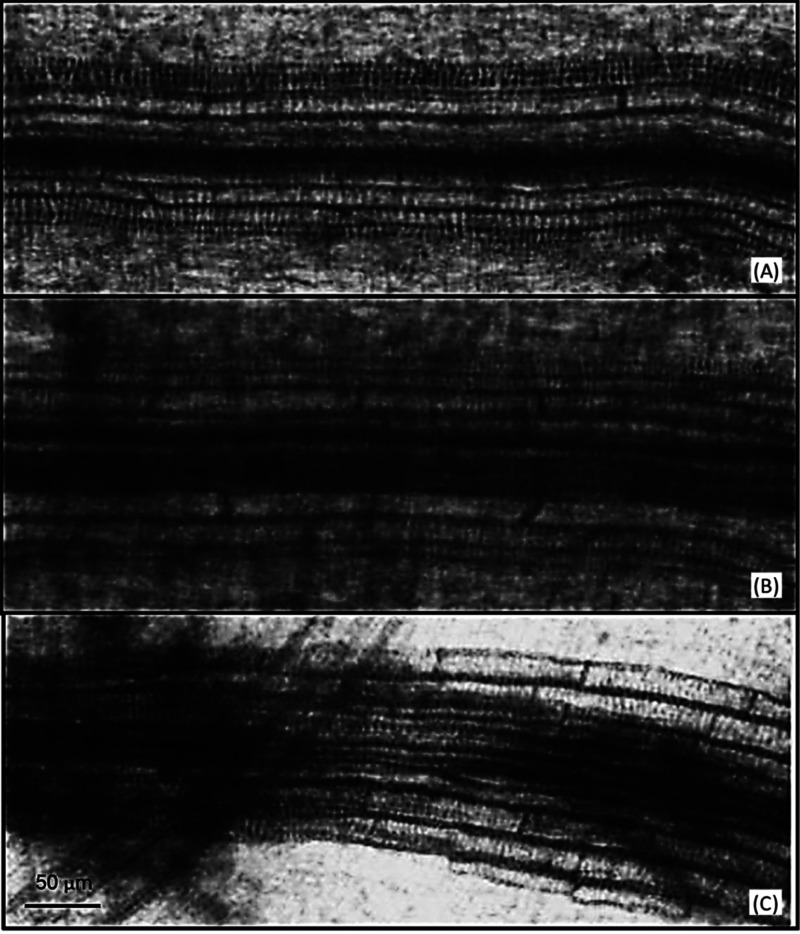
Photomicrographs of root metaxylem cells of *I. grandifolia* seedlings grown in the presence of (A) control and at concentrations of (B) 100 and (C) 1000 μM of compound **1** (piptocarphin A). Scale = 50 μm.

The evaluation of root anatomic aspects of the *I. grandifolia* allowed a detailed understanding of the action of piptocarphin A on the primary components of vascular tissue. In the primary growth, the metaxylem elements start their differentiation late and only complete their maturation after the elongation process has been completed. Therefore, they are less affected than the tissues around them.^53^ Beyond this, the presence of thick cell walls in the elements of the metaxylem makes it more rigid than the protophloem and also allows this tissue to be better observed as an anatomical character for analysis. Piptocarphin A strongly reduced the length of root metaxylem elements of *I. grandifolia*. Although in the control, the metaxylem elements reached an average size of 204 μm, in the seedlings grown under the action of piptocarphin A, the recorded sizes were 168, 93, and 72 μm, respectively, for concentrations of 100, 300, and 1000 μM (Figures 7 and 8).

From the data distributed as relative frequencies (%) of size class, it was found that in the control treatment, there was a homogeneous distribution of metaxylem element size, with the highest value of frequency (22.5%) found for elements belonging to the size between 200 and 240 μm (S6). Different from the control, in treatments with piptocarphin A, the predominant metaxylem elements sizes were 80–120 μm (40%) and 40–80 μm (70%), respectively, for concentrations of 300 and 1000 μM. No elements larger than 160 μm were recorded at the 1000 μM (S6). These results corroborate that piptocarphin A can inhibit root growth of *I. grandifolia* by interfering in the development of metaxylem vessel elements.

The results demonstrate that the inhibitory activity of *P. rotundifolia* leaf extract is due to the presence of piptocarphin A, the sesquiterpene lactone isolated in greater quantity and also the most active in this study. This study represents the first report on the phytotoxic activity of this compound in weed seed bioassays, hydroponic culture, and an anatomical study of metaxylem cells in which weeds with relevant agricultural importance were affected. This work represents one approach for the use of *P. rotundifolia* for weed control in agriculture by using its metabolites as natural herbicide leads. It also opens future perspective to investigate the presence of synergistic effects between the compounds to increase the bioactivity, as observed in the fractions. Also, the possible use of extract or enriched fractions for the formulation of natural herbicides is an interesting research that must be developed in the future.

## Supporting Information Available

Wheat coleoptile and phytotoxic bioassay protocols; effects of crude *P. rotundifolia* aqueous leaf extracts, the EtOAc and aqueous fractions and the sub-fractions obtained from the EtOAc fraction on wheat coleoptile elongation; cluster analysis of the phytotoxic activity of the compounds obtained from *P. rotundifolia* leaves on the germination and growth of weeds; and relative frequencies (%) of size classes of metaxylem cells from the roots of *I. grandifolia* seedlings grown with the control solution and in the presence of various concentrations of piptocarpin A (PDF)
