# Supplementary material for: Search of New Tools for Weed Control Using Piptocarpha rotundifolia, a Dominant Species in the Cerrado
Source: J Agric Food Chem. 2021 Jul 30;69(31):8684–94. doi: 10.1021/acs.jafc.1c01880 (PMC8638263; doi:10.1021/acs.jafc.1c01880)
Supplement: Supplementary file 1 — jf1c01880_si_001.pdf [file jf1c01880_si_001.pdf]

SUPPORTING INFORMATION

Search of New Tools for Weeds Control Using *Piptocarpha rotundifola*, a Dominant Species in the Cerrado

Simoni Anese,† Carlos Rial,‡ Rosa M. Varela,‡ Ascensión Torres,‡ José M. G. Molinilla,‡ Francisco A. Macías‡*

† Federal Institute of Education, Science and Technology of Mato Grosso - Campus Campo Novo do Parecis, MT 235 Km 12, Campo Novo do Parecis, MT 78360-000, Brazil

‡ Allelopathy Group, Department of Organic Chemistry, Institute of Biomolecules (INBIO). Campus de Excelencia Internacional (ceiA3), School of Science, University of Cadiz, C/ República Saharaui nº 7, 11510 Puerto Real, Cadiz, Spain

*(*Phone: +34 956012770; fax: +34 956016193; e-mail: famacias@uca.es)*

Number of pages: 7

S1. Wheat coleoptile bioassay protocol.

Approximately 100 wheat caryopses (*Triticum aestivum* L. cv. Catervo) were sown in 15 cm diameter Petri dishes (11 cm × 11 cm × 3.5 cm) lined with one sheet of qualitative filter paper and moistened with 10 mL of distilled water. The dishes were placed in a germination chamber for 4 days at 25±1 °C in the dark (Hancock, Barlow and Lacey, 1964). Following this period, the etiolated seedling coleoptiles were cut into 4 mm pieces with the aid of a Van der Veij guillotine under safe green light for use in bioassays. The fractions and purified compounds were pre-solubilized in dimethylsulfoxide (DMSO) and diluted with a buffered solution (pH 5.6) containing citric acid monohydrate (1.05 g L^{-1}), potassium hydrogen phosphate trihydrate (2.9 g L^{-1}) and 2% sucrose (Nitsch and Nitsch, 1956). The solutions containing the fractions were tested at concentrations of 0.2, 0.4 and 0.8 mg mL^{-1}, and the solutions containing the isolated compounds were tested at concentrations of 10 µM, 30 µM, 100 µM, 300 µM and 1 mM. A constant DMSO concentration of 5 µL/mL was maintained for each solution evaluated (Nitsch and Nitsch, 1956). Each treatment was tested in triplicate by adding 2 mL of each solution and five fragments of wheat coleoptile to a glass test tube (16 × 100 mm, 10 mL). One control containing the buffer solution plus DMSO (5 µL/mL) was performed. The commercial herbicide Logran, the original formulation of which is a combination of N2-*tert*-butyl-N4-ethyl-6-methylthio-1,3,5-triazine-2,4-diamine (terbutryn, 59.4%) and 1-[2-(2-chloroethoxy)phenylsulfonyl]-3-(4-methoxy-6-methyl-1,3,5-triazin-2-yl) urea (triasulfuron, 0.6%), was used as an internal reference, at the same concentrations and under the same conditions as reported previously. The test tubes were capped and kept in a growth chamber at 25±1 °C in the dark and with constant rotation in a drum tube rotator (0.25 rpm). After 24 h, the coleoptiles were removed from the tubes, photographed and measured. Data were analyzed statistically using Welch's test and are presented as percentage difference with respect to the control. Positive values represent stimulation, and negative values represent inhibition.

S2. Phytotoxic bioassay protocol.

The ethyl acetate fraction and the most active compounds were assessed for phytotoxic activity in the following eight species, which are agricultural weeds: morning glory (*Ipomea grandifolia*, Convolvulaceae); barnyard grass (*Echinochloa crus-galli*, Poaceae); slender amaranth (*Amaranthus viridis*, Amaranthaceae), guinea grass (*Panicum maximum,* Poaceae), brachiaria (*Uruchloa decumbens,* Poaceae*)*, perennial ryegrass (*Lolium perenne*, Poaceae), annual ryegrass *(Lolium rigidum*, Poaceae), capim-annoni (*Eragrostis plana*, Poaceae). For the bioassay, seeds from the recipient species were distributed in Petri dishes (5 cm diameter) lined with one sheet of Whatman N° 1 qualitative filter paper moistened with 1 ml of the extracts, compounds or control solutions, separately. The *I. grandifolia* seeds were first scarified for 5 min in a concentrated sulfuric acid solution and washed thoroughly with distilled water (Anese *et al.*, 2015).

Germination and growth were conducted in aqueous solutions at controlled pH by using 10^{-2} M 2-[*N*-morpholino] ethanesulfonic acid and 1 M NaOH (pH 6.0). The extracts or compounds to be assayed were dissolved in DMSO, and these solutions were diluted with buffer (5 µL DMSO solution/mL buffer) to give test concentrations for each extract (0.8, 0.4, and 0.2 mg mL−1) and compound (10 µM, 30 µM, 100 µM, 300 µM and 1mM). Parallel controls were also run as described above for the coleoptile bioassay.

The experimental design was randomized, with four replicates of 20 seeds per treatment. The experiment was conducted in a germination chamber at 25 °C with a 12 h photoperiod for 7 days for *I. grandifolia* (Anese *et al.*, 2015) and at 25 °C in the dark for the other species. Bioassays took 6 days for *E. plana, A. viridis*, *E. crus-galli, L. perenne*, *L. rigidum,* and 8 days for *P. maximum* and *U. decumbens*. After growth, plants were frozen at –10°C for 24 h to avoid subsequent growth during the measurement process. The germination rate, root length, and shoot length were recorded using a Fitomed system. Data were analyzed statistically using Welch's test, with significance fixed at 0.01

and 0.05. Results are presented as percentage differences with respect to the control. Zero represents control, positive values represent stimulation, and negative values represent inhibition.

S3. Effects of crude *P. rotundifolia* aqueous leaf extracts and the EtOAc and aqueous fractions on wheat coleoptile elongation. Values are expressed as percentage difference with respect to control. Each bar is the mean ± standard deviation. Significance levels $p < 0.01$ (a) or $0.01 < p < 0.05$ (b).

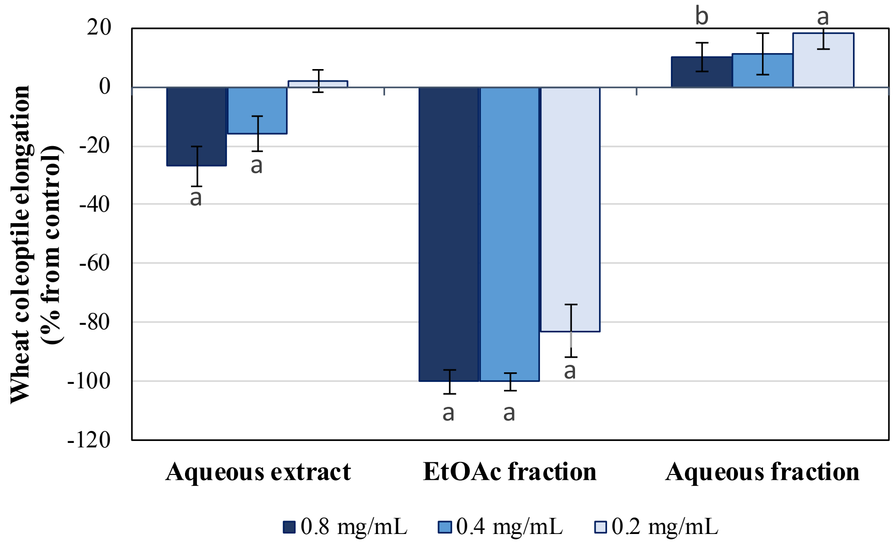

S4. Effect of the first four fractions obtained from the EtOAc fraction (A, B, C, and D) and the triasulfuron herbicide Logran (Lg) on wheat coleoptile elongation. Values are expressed as percentage difference with respect to control. Each bar is the mean ± standard deviation. Significance levels $p < 0.01$ (a) or $0.01 < p < 0.05$ (b).

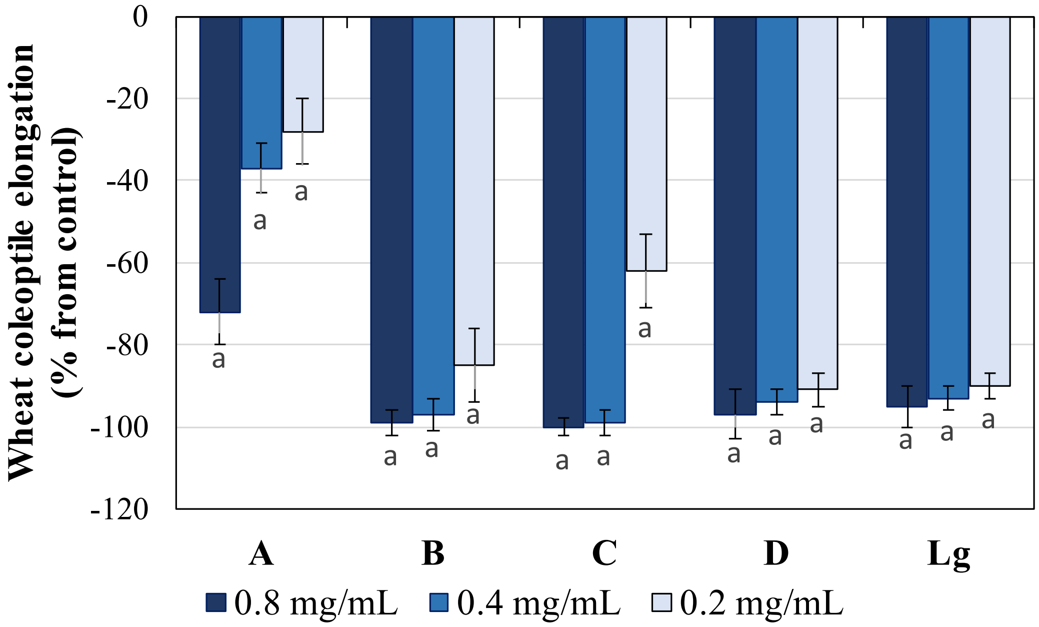

S5. Cluster analysis of the phytotoxic activity of the compounds obtained from *P. rotundifolia* leaves on the germination and growth of weeds.

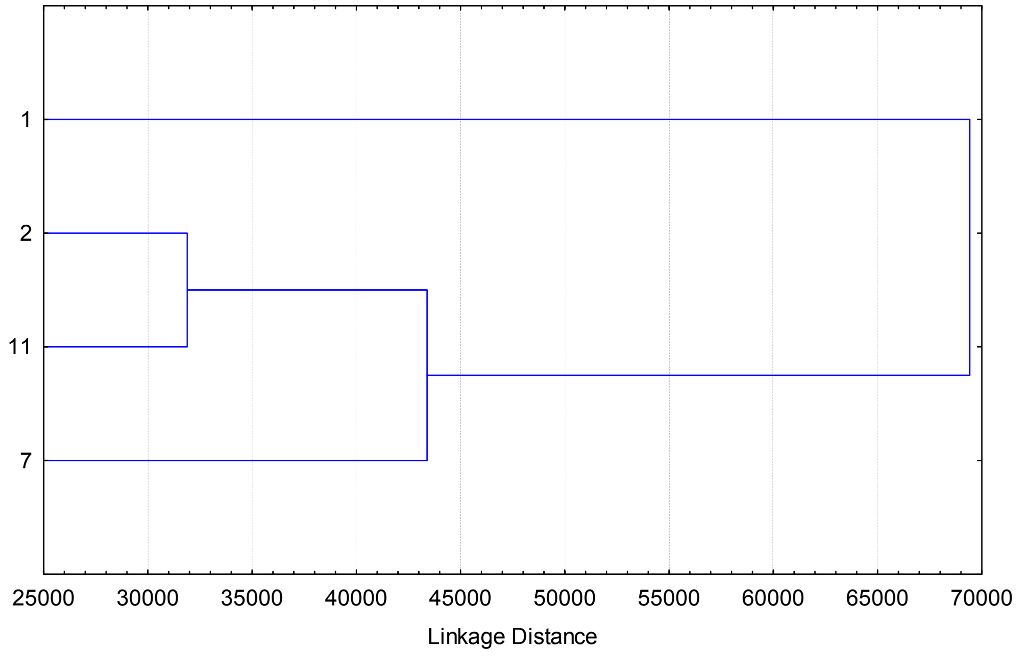

S6. Relative frequencies (%) of size classes of metaxylem cells from the roots of I. grandifolia seedlings grown with the control solution and in the presence of various concentrations of piptocarpin A.

**Size-class (µm)**	**0 (control)**	**10 µM**	**30 µM**	**100 µM**	**300 µM**	**1000 µM**
40–80	0	0	0	2.5	35	70
80–120	5	0	5	10	40	27.5
120–160	20	7.5	50	30	17.5	2.5
160–200	17.5	10	17.5	30	7.5	0
200–240	22.5	40	20	8	0	0
240–280	20	25	7.5	7.5	0	0
280–320	10	15	0	0	0	0
320–360	5	2.5	0	0	0	0
